# Real-Time Three-Dimensional Pedestrian Localization System Using Smartphones

**DOI:** 10.3390/s24020652

**Published:** 2024-01-19

**Authors:** Beomju Shin, Taehun Kim, Taikjin Lee

**Affiliations:** 1 College of Information Science, Hallym University, 1 Hallymdaehak-gil, Chunchein 24252, Gangwon-do, Republic of Korea; bjshin@hallym.ac.kr; 2 Augmented Safety System with Intelligence Sensing & Tracking, Korea Institute of Science and Technology, 5, Hwarang-ro 14-gil, Seongbuk-gu, Seoul 02972, Republic of Korea; taehun@kist.re.kr; 3 TJ LABS, 15F, 419, Teheran-ro, Gangnam-gu, Seoul 06160, Republic of Korea

**Keywords:** indoor localization, cloud platform, fingerprinting, pedestrian dead reckoning, smartphone

## Abstract

Robust and accurate three-dimensional localization is essential for personal navigation, emergency rescue, and worker monitoring in indoor environments. For localization technology to be employed in various applications, it is necessary to reduce infrastructure dependence and limit the maximum error bound. This study aims to accurately estimate the location of various people using smartphones in a building with a cloud platform-based localization system. The proposed technology is modularized in a hierarchical structure to sequentially estimate the floor and location. This system comprises four localization modules: course level detection, fine level detection (FLD), fine location tracking (FLT), and level change detection (LCD). Each module operates organically according to the current user status. The position estimation range is defined as a total of three phases, and an appropriate location estimation module suitable for the corresponding phase operates to estimate the user’s location gradually and precisely. When the user’s floor is determined by an FLD, the two-dimensional position of the user is estimated by an FLT module that tracks the user’s position by comparing the received signal strength indicator vector sequence and radio map. Also, LCD recognizes the user’s floor change and converts the user’s phase. To verify the proposed technology, various experiments were conducted in a six-story building, and an average accuracy of less than 2 m was obtained.

## 1. Introduction

With the popularization of mobile devices, the necessity and importance of location-based service (LBS) have become increasingly important [1]. The location of a smartphone is accurately estimated using a global navigation satellite system (GNSS) in outdoor environments [2]. Location solutions through GNSS are being used in various applications, such as vehicle navigation, sharing economy services, delivery logistics, and augmented reality entertainment. Various researchers are conducting studies to develop indoor positioning systems (IPS) with GNSS performance levels in terms of accuracy, availability, and cost-effectiveness; however, estimating a stable location in a complex indoor environment is challenging [3,4,5]. Accurate location estimation is possible when the line of sight between the GNSS satellite and the smartphone is secured in an outdoor environment. However, estimating an accurate location in an indoor environment is more challenging than that in an outdoor environment [6]. The indoor environment comprises various structures such as corridors, halls, and rooms. In addition, the structure of the indoor environment changes non-periodically, and it is expensive to install the infrastructure. In some cases, it may be difficult to install the infrastructure in the desired location. In addition, radio frequency (RF) signals in indoor environments are susceptible to multipaths, leading to IPS performance degradation. Because the inaccurate positioning accuracy of the IPS limits the scope of LBS applications, a robust IPS, regardless of the indoor environment and heterogeneous devices, is required.

The purpose of this study is to quickly estimate and track the user’s location in large-scale indoor environments such as hospitals, airports, and shopping malls. While ultra-wide band (UWB) [7] or angle of arrival (AOA) [8] systems are capable of accurately estimating user location, they are not suitable because they require separate expensive infrastructure installation and a user receiver to build the system in the above-mentioned environments. It is more economical and practical to utilize existing infrastructure, such as WiFi access points or BLE beacons, and it is essential to use smartphones for an indoor navigation system for general users [9]. A barometer is useful for estimating a user’s elevation, but since it requires calibration, it is challenging to apply it directly to an unspecified number of users. Instead, since smartphones already contain accelerometers and gyroscopes, as well as various RF chips, it is possible to accurately estimate the user’s indoor location through these sensors.

In this study, we propose an indoor navigation technology that accurately estimates the location of a three-dimensional (3D) user in a high-rise building. Here, the 3D position refers to the floor of the user, and the two-dimensional (2D) coordinates refer to the location on the floor. This paper presents our research on expanding the 2D location estimation system, previously developed by our team, into 3D. We propose a hierarchical structure-based 3D indoor pedestrian localization technology. Using this proposed technology, we developed an advanced localization algorithm by combining the pedestrian dead reckoning (PDR) [10,11,12] and received signal strength indicator (RSSI) vector sequences. The main feature of this technology is to narrow down and estimate the user’s location candidates in a high-rise building. The position range to be estimated was defined as one to three phases, and a position estimation module suitable for each phase was applied. The proposed system comprises four modules: course level detection (CLD), fine level detection (FLD), fine location tracking (FLT), and level change detection (LCD). CLD uses BLE’s service set identifier (SSID) and RSSI to estimate three candidate floors where the current user is expected. (In the text, BLE ID means SSID.) In FLD, the user’s RSSI vector sequence and radio map were compared, and the floor with the highest correlation was determined. In FLT, an accurate location is tracked using a surface correlation (SC) algorithm. SC is an indoor location estimation technology based on RSSI vector sequences previously developed by our team [13]. This technique generates a spatial distribution of RSSI by utilizing the trajectory estimated by PDR and the RSSI vector sequences and then estimates the location by comparing it with the radio map. The generated spatial distribution is called the user RSSI surface (URS), and SC tracks the position with the highest similarity when comparing the URS and the radio map. In addition, as LCD is periodically performed, when the user moves between floors using stairs or elevators, floor changes are recognized. If the user’s floor has changed, CLD is performed again to estimate the level at which the user moves. To prove the feasibility of the proposed technology, various experiments were conducted in a six-story building, and its excellent performance was confirmed. Below are the contributions of this study.

1. Through the implementation of a hierarchical, modularized localization system, we achieve rapid and precise user location estimation. Modules tailored to the user’s current state are dynamically executed, and their organic interconnection facilitates the gradual refinement of the user’s location range.
2. We establish dependable user location estimation using only a limited number of BLE modules. Specifically, within the FLD and FLT modules, we harness RSSI vector sequence data to deliver accurate user location even in environments characterized by extensive RSSI signal interference.
3. Leveraging a cloud platform featuring a hierarchical architecture, we have extended location services to a diverse population of smartphone users in a cost-effective and efficient manner.

The remainder of this paper is organized as follows: Section 2 presents a review of related works, Section 3 explains the proposed system and algorithm, and Section 4 presents the experimental results and performance analysis. Finally, conclusions are summarized in Section 5.

## 2. Related Works

**Figure 1:**
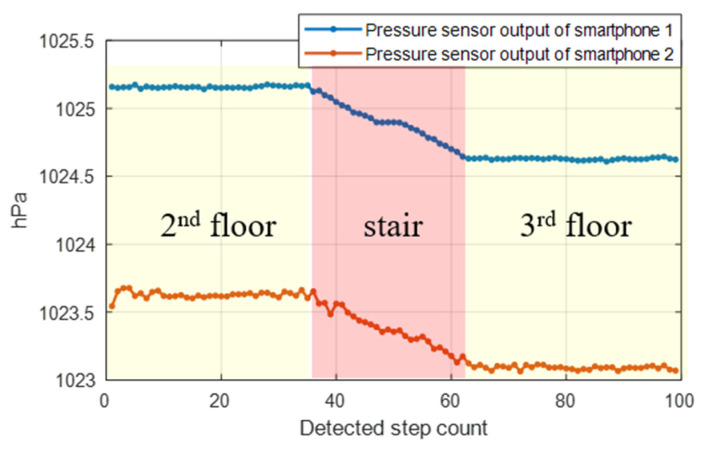
Changes in pressure sensor values of the two smartphones according to floor changes.

Accurately estimating a user’s floor in a high-rise building is extremely important information that can be used not only for LBS but also for emergency positioning [14]. Floor identification is the first step in target localization in multistory buildings. A study on floor estimation using a pressure sensor was conducted by researchers [15]. The pressure sensor responds immediately to the user’s altitude change, and its resolution is extremely high; therefore, it is possible to recognize sub-meter-level altitude changes. Because the pressure sensor includes a bias value, the user’s floor change can be recognized by the pressure sensor alone. However, to estimate the absolute altitude, calibration and external pressure information are required. Figure 1 shows the change in pressure when moving up one floor using two smartphones. As the altitude of the user increases, the air pressure decreases, and although the change patterns of the two smartphones are similar, a pressure difference occurs. In addition, the pressure value changed even when the pedestrian was stationary. In other words, this indicates that to estimate the altitude employed using the pressure sensor, its calibration and real-time sea level pressure information are required [16]. Consequently, these methods are difficult to apply universally because of the need for reference barometric pressure information and the need to pre-calibrate the pressure sensor in the user’s smartphone.

Studies on floor classification using RF signals are actively in progress [17,18]. Because WiFi AP and BLE beacons are installed on each floor of the building, the user’s floor can be determined using the RSSI signal pattern. In a complex indoor environment, the RSSI value is attenuated by walls, furniture, and people; however, it is mainly determined by the distance between the smartphone and transmitter. Estimating the AP installation floor with the highest RSSI signal as the current floor can sometimes lead to errors. The RSSI from the AP installed on a specific floor can be received sufficiently on other floors. In addition, the largest signal value among the RSSI vectors may be the signal from the AP installed on a different floor. Moreover, the RSSI vector values on adjacent floors tend to have similar patterns. To address this problem, Shao proposed multiple 3D autonomous blocks through a clustering algorithm based on WiFi fingerprints using RSSI and spatial similarity [19]. The same block contains fingerprints on different floors. Finally, the final floor is determined by calculating the joint probability for the autonomous block and local floor. A floor identification model based on linear discriminant analysis (LDA) was proposed by analyzing RSSI vectors from WiFi APs in an indoor environment [20]. They learned several LDA models that estimated one of the two floors and finally settled on the final floor by applying the voting method. A BLE RSSI-based adaptive weighted fusion algorithm is proposed to estimate the floor location for a multilevel atrium spatial environment [21]. In a multi-building multi-floor environment, extreme gradient boosting (XGBoost)-based indoor localization was proposed using a relational label that combines building ID and floor ID. XGBoost was used to learn a classification model that classifies floors and a regression model that estimates 2D coordinates.

After determining the floor, the localization system estimated the 2D location of the user. It is necessary to accurately track the location of the user for the LBS application or monitor the user’s location on the server. Typical indoor localization measurements include the time of arrival (TOA) [22], AOA [23], and RSSI [24]. RSSI is measurable in almost all RF communications. Because numerous RF infrastructures, such as LTE, WiFi, and BLE, are already installed indoors, it is possible to measure the RSSI of various RF signals. In addition, because LTE, WiFi, and BLE communication chips are already embedded in smartphones, users can use indoor navigation services without the need for separate infrastructure or receivers. Among the techniques for estimating the location based on RSSI, the most well-known technique is fingerprinting. Fingerprinting is a technique for estimating the RP with the best matching pattern as the current location by comparing the RSSI vector of the current user with that stored in the radio map. Fingerprinting technology must be preceded by radio map construction. The collector receives the RSSI pattern from the RP, and the coordinates and RSSI pattern of the corresponding RP are stored on the radio map. This technology does not require infrastructure or a receiver for localization but requires radio map construction because RSSI measurements are extremely noisy owing to the effect of multipath indoors, and the location error of the fingerprinting technology is generally larger than that of UWB and BLE AOA.

## 3. Proposed System

### 3.1. Architecture of Proposed System

**Figure 2:**
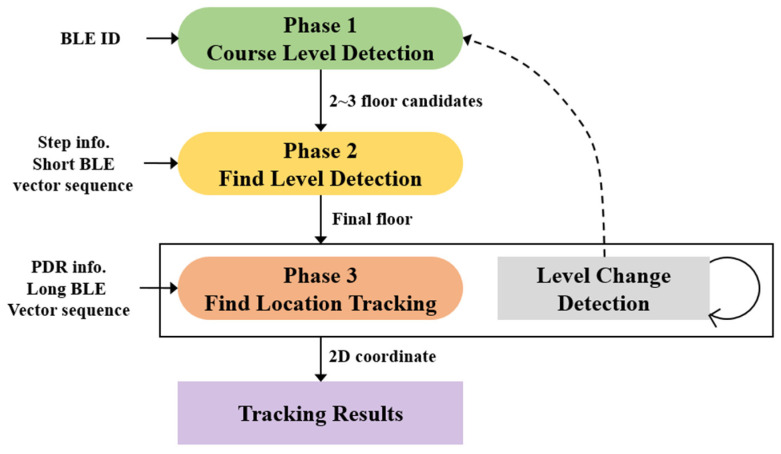
Proposed hierarchical localization system.

The purpose of this study was to accurately estimate the 3D location of a smartphone user in a high-rise building. Figure 2 shows the structure and process of the proposed system. In the proposed technology, each module is structured hierarchically. Each module uses different inputs to perform a specific function. In addition, the stages in which each module is executed are defined as Phases 1–3, and the user performs the localization request suitable for each phase. The proposed system estimates the user’s level and thereafter estimates the 2D coordinates while narrowing the user’s location candidate area. In addition, it periodically monitors the user’s floor change, and when a floor change occurs, it operates by finding the floor again. CLD estimates the candidate floors using the BLE IDs detected in the building. CLD selects two or three candidate floors where the user is likely to be. In CLD, a floor candidate group is estimated using only the BLE installation information for each floor. In FLD, the floor is estimated by comparing the user’s step information and the RSSI sequence with the radio map. The result of comparing the RSSI sequence and the radio map for each floor was calculated first, and the ratio of the RSSI difference values of the two floors with the highest correlation was calculated. A final floor was selected if the ratio exceeded the FLD threshold. In FLT, the current location of the user is continuously tracked using PDR and RSSI vector sequences. First, a user URS is created using the user’s PDR result and RSSI sequence, and the location is estimated by comparing the URS and the radio map. LCD detects that the user moves between floors using stairs or an elevator. LCD uses a BLE ID and RSSI to detect user movement between floors. LCD is performed periodically, and if the user’s floor change is recognized, CLD is performed again to estimate candidate floors.

### 3.2. Proposed System Implementation on Cloud

**Figure 3:**
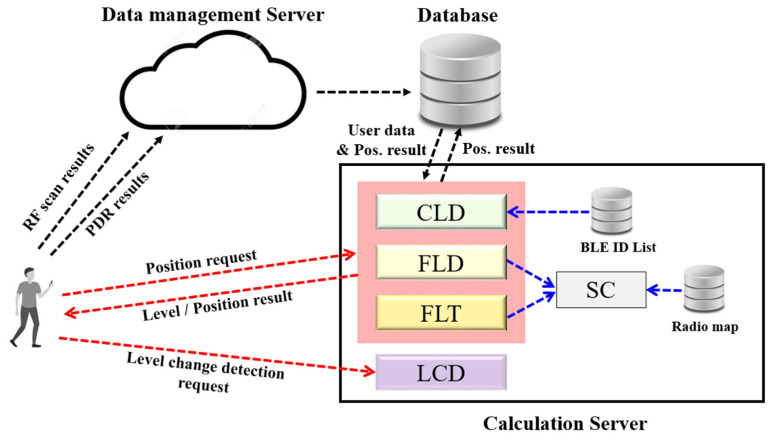
Proposed hierarchical localization system.

Figure 3 shows the data transmission and location request processes of the proposed system. The proposed system estimates the 3D positions of multiple users in real time using a cloud platform. It uses two servers. Separating the data management server that manages the transmitted user data from the location calculation server that responds to the user’s location request facilitates data management and responds to the user’s location request more quickly. Mobile devices transmit RF data and PDR results to the data management server. RF data were sampled every 0.2 s, and five BLE vectors were collected and transmitted every second. The PDR results comprise the step index, step length, and heading, and five sets of PDR results were collected and transmitted. The data management server stores the data received from multiple users in the database. In the calculation server, a position estimation module suitable for the current phase is performed for each user. As described, there are four localization modules in the calculation server. Each module was executed according to the user’s conditions. In addition, the BLE installation table, which stores information on the BLE installed on each floor, and the radio map, which stores RSSI spatial patterns, are also included in the server.

## 4. Explanation of Proposed System

### 4.1. Course Level Detection Algorithm

CLD estimates the current floor of the user by using only the received BLE ID. The purpose of CLD is to quickly approximate the user’s floor in a building. We already have a list of BLE modules installed on each floor. If most of the BLE modules installed on a specific floor are received, it can be assumed that the user is on that floor. However, even if BLE beacons installed at a distance on the same floor are not received, BLE beacons close to the user’s current location but on a different floor can be received. Considering these characteristics, the CLD module calculates the BLE reception probability for each floor using the list of BLE beacons installed on each floor and the received BLE ID. It then determines that the user is near the floor with the highest reception probability.

**Figure 4:**
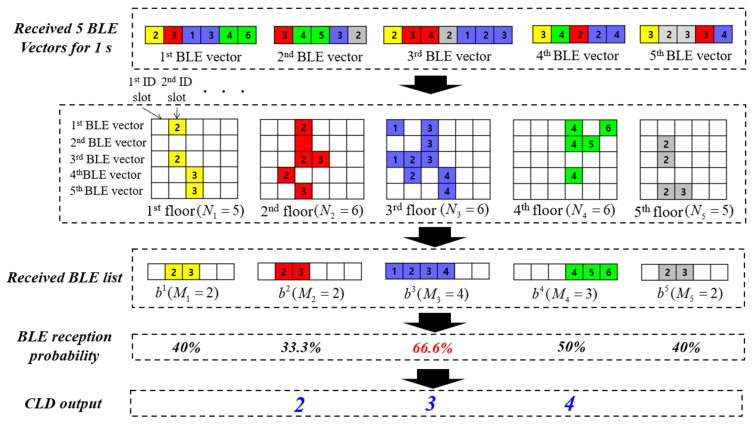
Creation of BLE list for CLD. CLD estimates a present user floor using only BLE vector for 1 s. CLD calculates the BLE reception probability of each floor and determines three candidate floors.

The ID of the BLE beacon installed on the ith floor is stored in the BLE installation table as follows:

(1) $$B^{i}=\{ID_{1}^{i},ID_{2}^{i},\ldots ,ID_{n}^{i},\ldots \}$$

where $ID_{n}^{i}$ denotes the nth BLE beacon ID installed on the ith floor. Figure 4 illustrates the CLD process. Identical colors represent BLE modules installed on the same floor. In the CLD module, the BLE vector received over a 1 s duration is utilized. In the proposed system, since the BLE vector is sent to the cloud server every 0.2 s, five BLE vectors are queried from the server. These vectors are then sorted by floor, and user BLE lists are generated for each floor. If a particular BLE beacon is detected even once out of these five times, it is marked as received. The received BLE list for the ith floor is expressed as follows:

(2) $$b^{i}=\{id_{1}^{i},id_{2}^{i},\ldots ,id_{k}^{i},\ldots \}$$

where $id_{k}^{i}$ denotes the kth scanned BLE ID installed on the ith floor. We create a user BLE list for all floors. Through these user BLE lists, it is possible to determine how many BLE beacons installed on each floor have been received by the user. For example, if the user receives signals from most of the beacons on the third floor but only from one beacon on the fifth floor, it is likely that the user is on the third floor. However, since the number of installed BLE beacons may vary from floor to floor, the following calculations are conducted to determine the reception probability for the BLE beacons on each floor:

(3) $$P_{B}=\{\frac{M_{1}}{N_{1}},\frac{M_{2}}{N_{2}},\ldots ,\frac{M_{i}}{N_{i}},\ldots \}$$

where $M_{i}$ denotes the number of overlapping BLE beacons of ith floor. The final estimated floor using only the BLE ID is as follows:

(4) $$E=\underset{i\in \{1,2,\ldots ,L\}}{\arg\max}(P_{B})$$

where *E* is the estimated floor with the highest probability of BLE reception, and L represents the highest floor of the building. BLE beacons’ range covers not just their installation floor but also adjacent ones. It is not guaranteed that the reception probability is highest everywhere on a specific floor. Thus, CLD reports the floor with the top reception probability, as well as the floors directly above and below it as follows:

(5) $$F_{CLD}=\left\{\begin{array}{c} 1,2\\ L-1,L\\ E-1,E,E+1 \end{array}\begin{array}{c} if\;\;E=1\\ else\;\;if\;\;E=L\\ otherwise \end{array}\right.$$

*F_CLD_* represents the set of candidate floors estimated by the CLD module. The CLD module estimates multiple floors rather than a single floor, quickly determining the floors where the user is likely to be currently located. If the BLE reception probability is highest on either the first floor or the top floor, then two floors are determined. However, if the estimated value of E falls within *1* < *E* < *L*, three floors are determined by adding the floor above and below the estimated floor. Subsequently, FLD uses the RSSI spatial vector to determine one floor from the *F_CLD_*.

### 4.2. Fine Level Detection Algorithm

**Figure 5:**
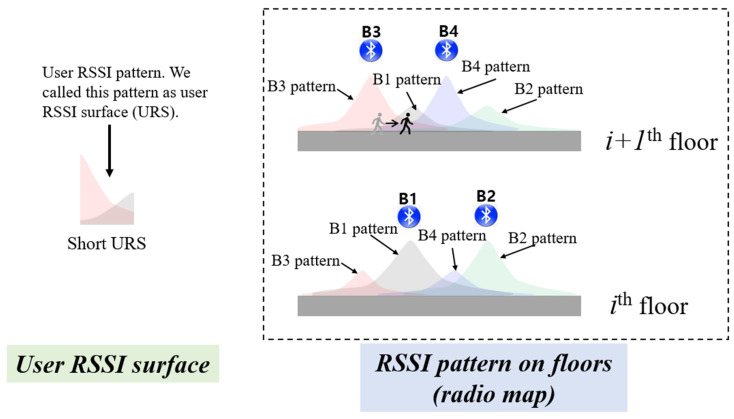
The beacon signal can reach many floors, but it is strongest on the floor it is installed. FLD uses a short RSSI sequence to find out which floor the user is on.

**Figure 6:**
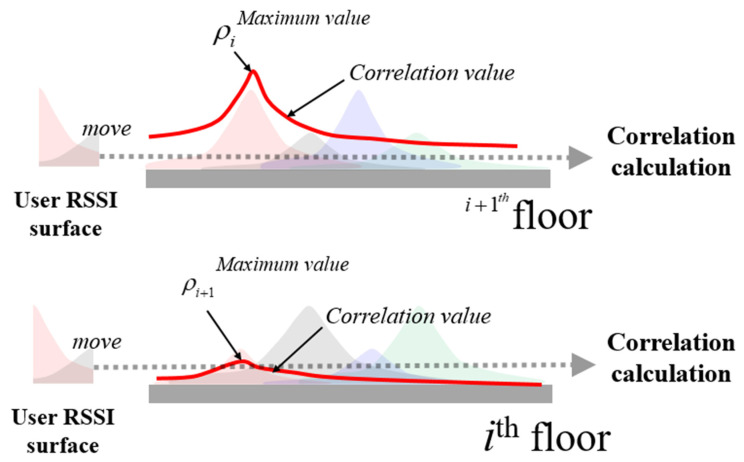
The CLD module offers a rough estimation of the user’s floor level. Subsequently, the FLD refines and finalizes the floor determination by leveraging short URS among the CLD outputs. Specifically, FLD identifies the best correlation position between the URS and the radio maps from CLD outputs.

In CLD, only the ID of the received BLE signal is utilized. In contrast, FLD employs both the RSSI value and the ID for more accurate floor determination, akin to traditional fingerprinting methods. Rather than relying on a single RSSI vector, FLD uses RSSI vectors received over 5 s. Its approach closely resembles that of FLT from the next subsection. However, instead of using the user’s actual trajectory, FLD converts the 5-s RSSI vector into a 5-m-long trajectory for correlation computation. Figure 5 illustrates the concept of FLD, assuming that each floor is a one-dimensional space and two beacons are installed per floor. The strongest RSSI typically comes from the beacon on that particular floor, but signals from beacons on adjacent floors can also be received. For example, while B3 is placed on the *i +* 1th floor, its signal might also be detectable on the ith floor. FLD works by calculating the cross-correlation between the user’s RSSI pattern and the established radio map pattern. An example of this cross-correlation with user RSSI patterns and individual floor radio maps is depicted in Figure 6.

To determine the user’s current floor, FLD examines the maximum correlation value for each floor. The outcome obtained by computing the correlation between the user’s RSSI pattern and the radio map is denoted as the surface correlation coefficient (SCC). If the ratio between the maximum and the second-highest correlation value surpasses the FLD threshold, the floor with the top correlation is identified as the user’s current floor. The vector of the maximum correlation values for the two floors in Figure 5 can be expressed as follows:

(6) $$C_{F}=[\begin{array}{cc} \rho_{j} & \rho_{j+1} \end{array}]$$

where $\rho_{i}$ is the maximum correlation value of ith floor radio map. The final user floor, *F*, is estimated according to the maximum correlation value ratio between floors as follows:

(7) $$F=\left\{\begin{array}{cc} j & if\frac{\rho_{j}}{\rho_{j+1}}>FLD_{threshold}\\ 0 & otherwise \end{array}\right.,$$

where $\rho_{i}$ and $\rho_{i+1}$ denote the largest and second largest value among vector $C_{F}$ , respectively. When the ratio surpasses the FLD threshold, the user’s floor is confirmed. Upon this determination, the FLT module activates, utilizing the user’s RSSI vector sequence and PDR trajectory to precisely track the user location.

### 4.3. Surface Correlation

While RSSI is easily measurable, it has a very noisy characteristic indoors, making it very challenging to accurately estimate a user’s location using only RSSI. To address this issue, we developed the FLT module utilizing the RSSI vector sequence. Specifically, the algorithm used in the FLT module is SC. For a detailed description of this algorithm, refer to [9]. In this text, we will briefly discuss the principle of SC.

**Figure 7:**
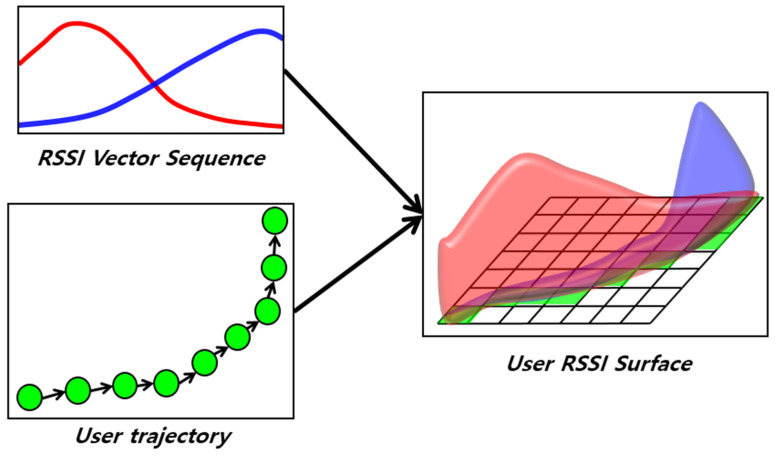
SC uses the RSSI pattern in the space where the user moved. This is called URS, and URS is created using the user’s trajectory and RSSI vector sequence.

**Figure 8:**
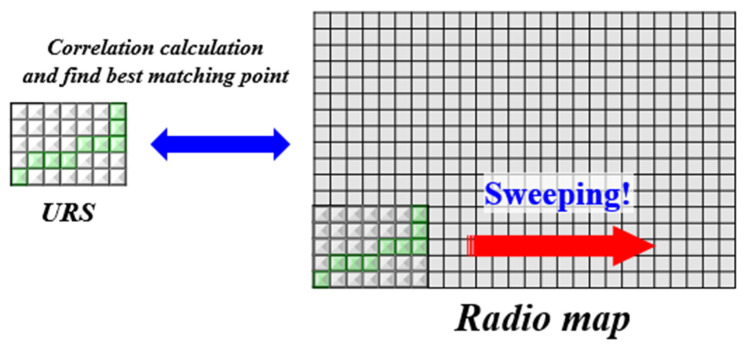
SC finds the location on the radio map that has the most similar similarity to URS and that location becomes the current user’s position.

SC operates based on the fingerprinting method. That is, it compares the user’s RSSI pattern with a radio map and considers the position with the highest similarity as the current location. SC does not simply estimate the location using a single RSSI vector; instead, it uses accumulated RSSI vectors to determine the location. It synchronizes the user’s trajectory with the RSSI vector sequence to create an RSSI distribution in space. Figure 7 depicts the creation of URS using the PDR trajectory and the RSSI vector sequence. The URS includes the spatial distribution of RSSI. SC calculates the similarity between the URS and the radio map and takes the position with the highest correlation value as the current location. Figure 8 illustrates the operation process of SC. It moves the URS over the radio map to find the optimal position with the highest correlation. In traditional fingerprinting methods, user data are merely a single vector. The location is determined by calculating the similarity with the radio map and selecting the RP with the smallest difference in the RSSI pattern. The proposed method creates user data in the form of a surface and computes their correlation with the entire radio map. This is why it has been named ‘surface correlation’. By aggregating RSSI to determine the location, it enhances spatial discernment and provides accurate location results even in noisy indoor environments.

### 4.4. Level Change Detection Algorithm

LCD is a module that quickly recognizes when a user moves between floors using stairs or an elevator. The purpose of LCD is not to estimate the floor as CLD or FLD but to quickly recognize the user’s floor change. Because the CLD, FLD, and FLT modules are executed according to the user phase, LCD is not executed simultaneously. LCD is performed periodically to quickly recognize the user’s floor movement. Generally, FLT is performed until the user moves to the next floor. The radio map used in FLT stores not only the BLE beacons installed on the current user’s floor but also the BLE patterns installed on other floors. Therefore, even if movement occurs between floors, the SCC value does not decrease rapidly. If there is no LCD module, there is a possibility that a long delay may occur in determining the floor movement. LCD is similar to the interrupt concept, and when it recognizes the movement between floors quickly, it performs CLD.

**Figure 9:**
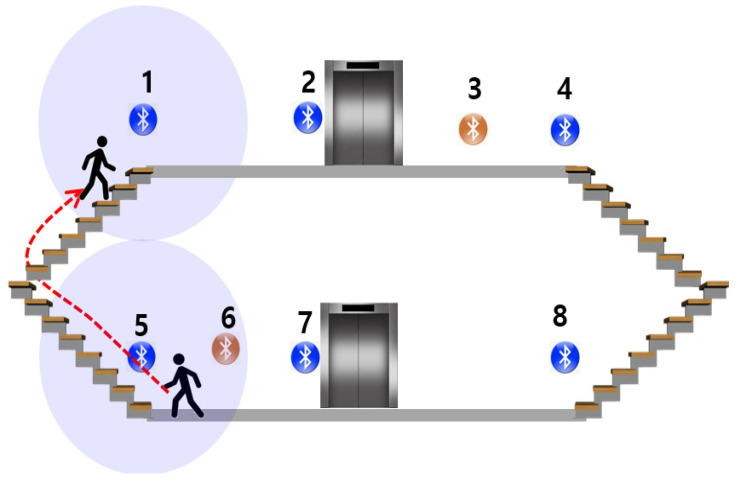
LCD module principle. The LCD module monitors the current user’s floor and the maximum value of the current user’s RSSI pattern and detects the user’s movement between floors.

LCD relies on the strength of the BLE signal installed around stairs or elevators. Figure 9 illustrates the principle behind the LCD process. We assumed the installation of eight BLE beacons. While all beacons are utilized in the FLT, only a specific beacon (indicated in blue) is utilized in the LCD. LCD periodically monitors the blue beacons. If the user is downstairs, one of the blue beacons (5, 7, or 8) will have the maximum signal strength. When the user ascends using the left staircase, the RSSI value of beacon 1 reaches its peak. If a beacon installed on a different floor than the one determined through FLD registers the maximum signal value, LCD transitions the user’s state into the CLD phase.

### 4.5. Implementation

The proposed system aims for real-time navigation and tracking services. To this end, we applied the Google Cloud platform (GCP) for the real-time experiment. The proposed system comprises a mobile application, database management server, and calculation server. The mobile application sends measurements to the database management server and, depending on the phase, sends position requests to the calculation server. GCP provides several products, and among them, the system was configured using Cloud Structured Query Language (SQL) and Cloud Run. Cloud SQL is a relational database that stores data sent from mobile phones or the results computed by a Cloud Run. Communication with the smartphone, data parsing, and location calculations were performed in Cloud Run. The CLD, FLD, or FLT modules are executed in Cloud Run according to the user’s request, and the calculated position result is transmitted to the user and stored in the database.

## 5. Experimental Results

### 5.1. Experiment Setting

**Figure 10:**
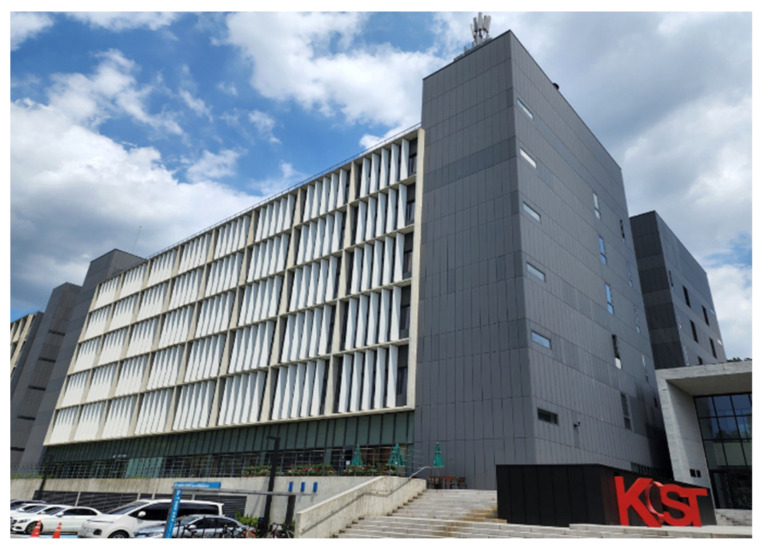
KIST L3 building for testbed.

**Figure 11:**
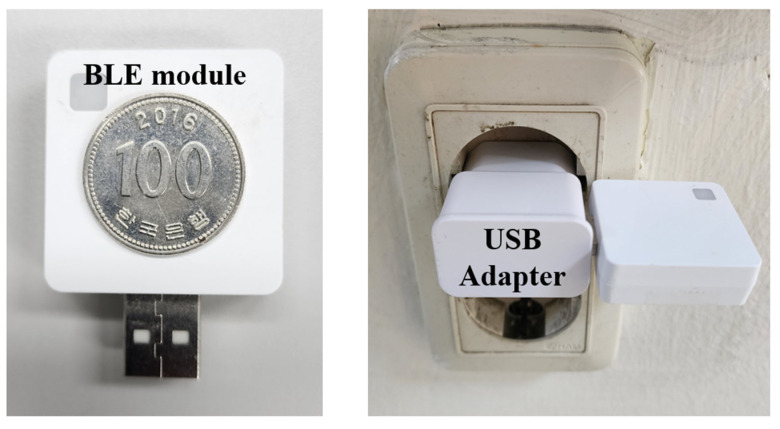
USB-type BLE beacon. It can be easily installed by connecting it to a battery or an adapter.

**Figure 12:**
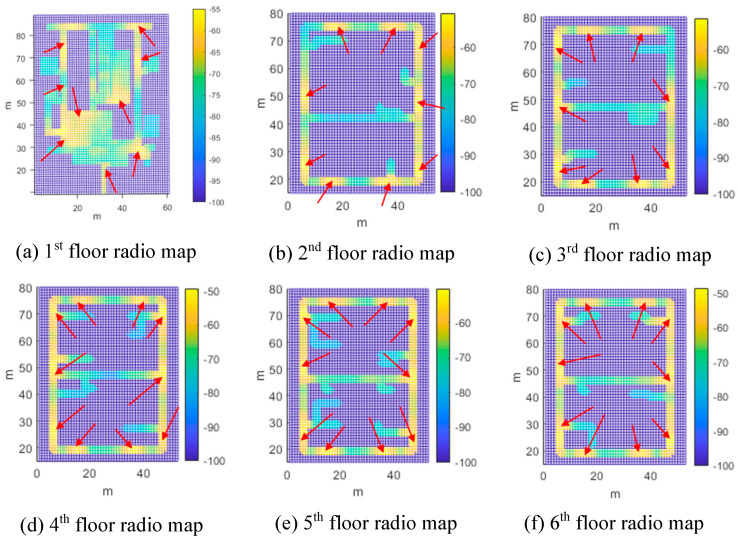
Radio map of each floor. Red arrows indicate the beacon installation positions.

To evaluate the performance of the proposed system, various experiments were conducted on the six-story Korea Institute of Science and Technology (KIST) L3 building, as shown in Figure 10. The first floor of this building comprises a lobby, rooms, and corridors, and the second to sixth floors comprise research rooms and corridors, respectively. There were also two elevators and four stair entrances on each floor. The ceiling height of the building was approximately 3.5 m. A total of 57 self-made USB-type BLE beacons were installed in the testbed building. Figure 11 shows a USB BLE beacon that can be easily installed indoors with an adapter or battery. Figure 12 shows radio maps of the entire floor. The red arrow indicates the position of the beacon. In the radio map, the RSSI patterns in 2D space are stored for each beacon. In addition, the installed beacon lists for CLD and LCD are stored. Table 1 lists the installed beacon IDs on each floor.

We applied a radio map construction approach in our previous study. To build a radio map, we installed BLE beacons in a designated socket, carried a smartphone, and walked through all floors. In L3 buildings, it took less than an hour to install infrastructure and collect data, and it took less than 3 h to process data and generate radio maps. The Galaxy Note 20 was used to construct the radio map.

### 5.2. Experimental Results

An experiment was conducted to evaluate the performance of each proposed module on the testbed. The CLD experiment was conducted 10 times at the entrance to the stairs on each floor or near the elevator. The FLD experiment was conducted at the same location as the CLD request. To confirm the performance of FLT, a closed-loop path scenario was applied on the first floor. In addition, to verify the actual real-time location tracking performance, an experiment was conducted to move several floors using stairs and elevators. The performances of LCD, FLD, FLT, and interfloor movement detection module, LCD, were confirmed. The true data used in the experiment were acquired through pedestrian heading correction and map-matching. Galaxy Note 20 (D1) and Galaxy 22 Ultra (D2) smartphones were used for experimental data logging.

#### 5.2.1. CLD Results

**Figure 13:**
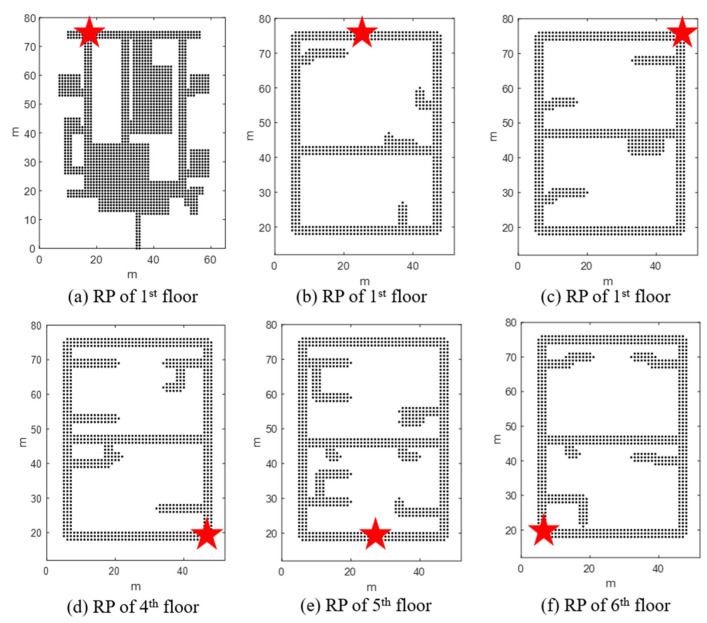
CLD request position of each floor. The red stars represent the CLD experiment location.

If the user phase is one, the CLD module is operated. CLD is an algorithm that finds a user’s floor using only the IDs of the nearby BLE beacons received from a smartphone. Because it is difficult to accurately estimate the user’s floor with only the BLE ID, two or three floor candidates are determined. CLD uses shot scan data and quickly determines the floor. Figure 13 displays the CLD request positions on each floor. A total of 10 CLD tests were performed at corresponding locations on each floor. CLD calculates the probability that a user is on each floor based on the received BLE ID. Under the premise that the user receives the BLE module installed on the current floor the most, the probability is calculated using the BLE beacon information installed on each floor and the currently received BLE ID vector.

Table 2 lists the CLD results for each device. The result for each floor was the last result of the 10 CLD requests. All current floors were included in the CLD results. In other words, it succeeded in estimating the current user’s floor in a course manner using only the BLE ID. In addition, the maximum probability floor may differ from the current floor. (If the probability is the same, it is indicated as a lower floor.) This also implies that it is difficult to accurately determine a user’s floor using only the BLE ID. Therefore, only the BLE ID is used to infer the floor where the current user is likely to be present, and then one floor among the CLD results is determined using FLD using the RSSI vector sequence. Table 3 summarizes the CLD results. The maximum probability floor is indicated in bold.

From Table 3, we observe that all the CLD requests include the current floor. In the case of D2, all current floors have the maximum probability. The number of BLE IDs scanned is different for each device; however, in terms of CLD performance, a device with a smaller number of BLE IDs scanned exhibits almost identical performance.

#### 5.2.2. FLD Results

CLD estimates the three floors using only the received BLE ID. When CLD estimates the candidate floors, the user phase increases to 2. Finally, FLD determines one of the three floors. FLD utilizes the RSSI vector received for 5 s to generate a 5-m-long URS without using the actual trajectory. The ratio between the floor with the largest SCC value and that with the second-largest SCC value was calculated, and the final floor was fixed when the corresponding value exceeded a certain threshold. FLD experiments were also conducted at the locations shown in Figure 13 for all floors. Before the FLD operation, CLD was performed first, and FLD was performed using the CLD results and short URS. (The FLT experiment was performed after the last test in the CLD scenario.) Table 4 summarizes the results of the FLD experiments.

**Figure 14:**
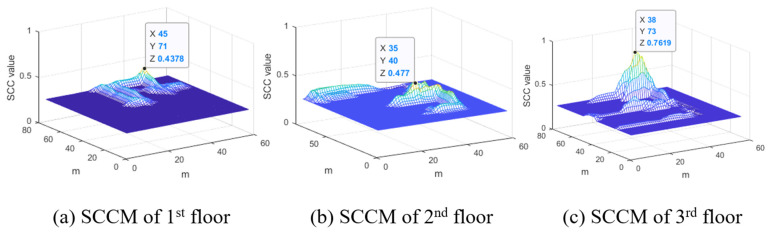
FLD results on the third floor using D1. The candidate floor from CLD are first, second, and third floors.

SCC represents the degree of similarity between the short URS and radio map. In particular, although the maximum probability of CLD on the third and fourth floors of D1 was incorrectly estimated for the second and fifth floors, respectively, it was confirmed that the floors were accurately estimated through FLD. This shows that the coverage of the BLE beacon reaches several floors of the building; however, the current user floor is clearly estimated through the RSSI. Figure 14 shows the SCCM calculated by performing FLD on the third floor using D1. The first, second, and third floors were returned through CLD calculations. The maximum value of SCCM calculated after comparing the radio map and short URS of each floor is shown in Figure 14. Because the ratio between the maximum SCC value of the third floor and the maximum SCC value of the second floor is higher than that of the FLD threshold, the current user floor of the building is fixed to the third floor, and the user phase is updated to the next phase.

It also maximizes computational efficiency by using CLD and FLD together. For instance, in dozens of high-rise buildings, there is excessive computation to directly apply FLD. Therefore, after estimating the approximate floors through CLD, it is estimated through FLD. In addition, the exact floor can be estimated by generating the RSSI spatial pattern through the RSSI vector sequence and comparing it with a radio map.

#### 5.2.3. Multi-Floor Movement Test Result

**Figure 15:**
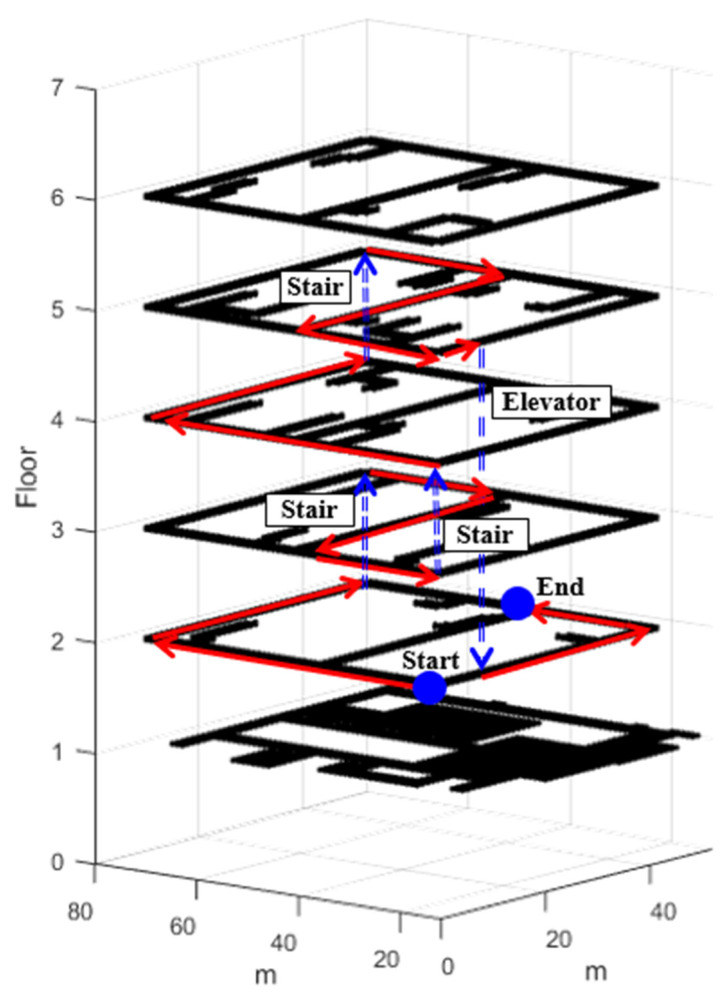
Multistory pedestrian tracking test scenario. The red line represents movement on the same floor, and the blue dotted line indicates movement between floors.

To verify the performance of the proposed technology in a multistory building, we performed a test, as shown in Figure 15. The experimenter moved from the second to the fifth floor through the stairs and moved from the fifth to the second floor using the elevator. When a user moves between floors, the floor change is immediately determined using an LCD.

**Figure 16:**
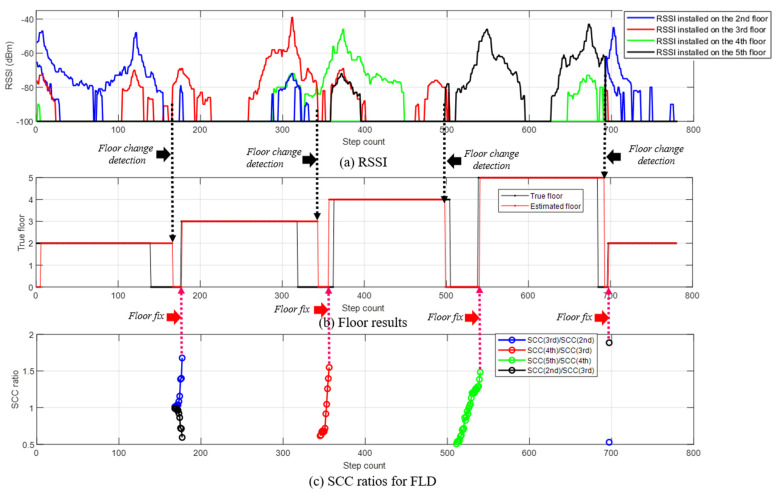
Floor estimation results. LCD detects the user movement between floors by checking the current floor and maximum RSSI signal. FLD calculates the SCC and determines the floor when the SCC ratio exceeds the FLD threshold.

**Figure 17:**
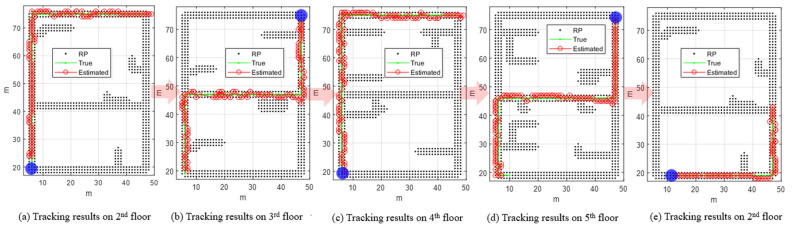
FLT results of the multistory test scenario. Red circles indicate the FLT result and blue circles are the starting points of each floor.

The floor is estimated through CLD and FLD, and the fine location of the pedestrian is tracked through FLT. Figure 16 shows the user floor estimation results. In Figure 16b, the black line represents the true floor result, and the red line represents the estimated floor result. In the black line, the label is indicated as 0 in the section where the user moves between floors, such as stairs or elevators. LCD detects floor changes using the RSSI strength of a specific BLE beacon. As shown in Figure 16a, LCD periodically monitored whether the signal strength of a specific BLE signal installed on another floor was greater than that of the BLE signal installed on the estimated current floor. When a floor change is detected, CLD estimates the candidate floors that the user is likely to be in and thereafter determines the final floor through FLD. Floor determination occurred when the SCC ratio exceeded the FLD threshold, as shown in Figure 16c. Except when moving from the fourth floor to the fifth floor, floor movement was detected through LCD when the user climbed more than half of the stairs. Because the BLE beacon is not installed around the stairs, there is a delay in detecting floor change; however, if BLE is additionally installed on the stairs, quick floor change detection will be possible. In addition, it is important to quickly determine the floor and perform FLT when the user moves to the next floor. Table 5 lists the number of steps at the time of completion of the true floor conversion and the number of steps at the time of completion of the estimated floor conversion. In addition, the time corresponding to each step when the experiment start time was 0 s is marked. The results demonstrate that there is a slight delay in detecting a floor change, but the next floor is quickly determined after detecting a floor change. Figure 17 shows the FLT performance on each floor. The green dots represent the true positions, and the red circles indicate the FLT results. The blue circles represent the starting points on each floor. It can be confirmed that the location of the pedestrian is extremely stably estimated using FLT.

**Figure 18:**
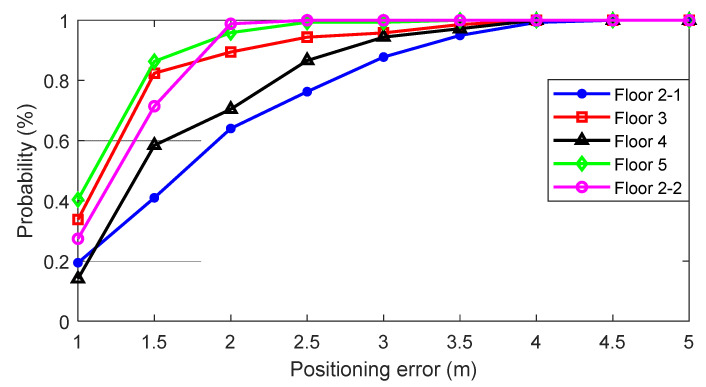
Cumulative density function of FLT results on each floor.

Figure 18 shows the cumulative density function of FLT results on each floor. Through this graph, it can be seen that FLT accurately tracks the user’s location. It shows that the location error converges to less than 3 m with a 90% probability on all floors. Table 6 summarizes the positioning performance analysis results for each floor.

### 5.3. Discussion and Future Work

The proposed technology initially determines the user’s floor within a multi-story building and subsequently calculates the 2D coordinates within that floor. This technology, based on fingerprinting, efficiently determines the user’s location by narrowing down the range of radio maps to be compared, starting with floor identification. The system estimates the user’s location by utilizing BLE signals. An advantage of using BLE RSSI lies in its compatibility with both iOS and Android smartphones, as iOS devices do not grant access to the RSSI measurement of WiFi access points.

Creating radio maps can be effectively achieved through methods such as cloud sourcing or Simultaneous Localization and Mapping (SLAM) [25], though ensuring consistent location performance can pose challenges. Efficiently creating and maintaining radio maps in large indoor spaces will continue to be a subject of ongoing research in the future. Swiftly and accurately creating a radio map and defining and managing its format for rapid location estimation will be very important. Additionally, the RSSI of a smartphone varies depending on the user’s direction indoors. Therefore, if the radio map stores RSSI values for various directions and utilizes them to estimate the user’s location more accurately, it would enable the provision of a more stable indoor navigation solution to users. In our proposed system, we have developed a USB-type BLE beacon designed for straightforward installation, directly managed within the testbed. While there is an associated infrastructure installation cost, the installation process is streamlined and expeditious, ensuring uniform performance across all testbed regions. Additionally, the SC technique leverages accumulated signals to achieve exceptional performance, even when the number of received BLE signals at the current location is limited.

In fingerprinting technology, discrepancies in RF sensitivity among heterogeneous devices can impede positioning accuracy. To address this concern, previous studies have utilized techniques such as RSSI difference [26] or calibration approaches [27]. In future SC methods, we aim to develop a feedback loop that enhances the correlation value between URS and the radio map to address this issue. We’ll define an RSSI bias for calibrating each smartphone’s RSSI sensitivity and estimate these biases to boost the correlation value. Applying device-specific RF sensitivity adjustments will enable us to rectify RSSI discrepancies across smartphones, thereby significantly improving indoor positioning performance.

## 6. Conclusions

In this study, we propose a hierarchical structured localization system that estimates a user’s 3D location in a multistory building. The proposed system gradually estimates the location of the user in the order of candidate floors, final floor, and 2D location, and each stage is defined as Phases 1 to 3. The proposed system comprises CLD, FLD, FLT, and LCD modules, and the corresponding modules operate according to the user phase. When a user initially enters a building, candidate floors are estimated through the CLD module using the received BLE ID as an input. Next, the current floor of the user was determined through FLD. This module compares the short RSSI vector sequence with the radio map of the candidate floors and determines the final floor when the ratio between the SCC values satisfies a specific condition. When the user’s floor is determined, the precise location of the user is tracked using the FLT module. FLT generates the URS using the user’s trajectory and RSSI vector sequence and thereafter estimates the user’s 2D location using the SC algorithm. The LCD module periodically monitors the user’s movement between the floors. When a user moves between floors using stairs or an elevator, this movement is detected through LCD. At this time, the user’s location is estimated by operating again sequentially from CLD according to the phase. The stability of the system was verified through various experiments, and it is expected that the proposed system will efficiently and accurately estimate a user’s location in a high-rise building.

The proposed technology is expected to find wide-ranging applications in rapidly determining a user’s 3D location in various indoor environments. Additionally, we plan to develop seamless navigation technology in the future, which will integrate pedestrian and vehicle navigation technologies, ensuring seamless transitions from outdoor to indoor environments.

## Figures and Tables

**Table 1:** BLE installation information.

Floor	BLE ID	The Number of Installed Beacons
1	1,2,3,4,5,6,7,8,9	9
2	10,11,12,13,14,15,16,17,18	9
3	19,20,21,22,23,24,25,26,27	9
4	28,29,30,31,32,33,34,35,36,37	10
5	38,39,40,41,42,43,44,45,46,47	10
6	48,49,50,51,52,53,54,55,56,57	10
The number of total installed beacons		57

**Table 2:** CLD performance per device (result of the 10th CLD request).

	Floor	Received Beacon ID	Floor of Maximum CLD Probability (%)	CLD Results (Bold: Maximum CLD Probability Floor)
D1	1	3,5,10	1 (22.2%)	**1**,2
	2	4,5,10,11,12,18	2 (44.4%)	1,**2**,3
	3	11,12,21,22	2 (22.2%)	1,**2**,3
	4	30,31,39,40,41	5 (30%)	4,**5**,6
	5	34,42,44,45,53	5 (30%)	4,**5**,6
	6	44,45,53,55,56,57	6 (40%)	5,**6**
D2	1	5,10	1 (11.1%)	**1**,2
	2	4,10,11,12	2 (33.3%)	1,**2**,3
	3	11,12,21,22,30	3 (22.2%)	2,**3**,4
	4	31,39,40,41	4 (20%)	3,**4**,5
	5	44,53	5 (10%)	4,**5**,6
	6	44,45,53,55,56,57	6 (40%)	5,**6**

**Table 3:** Total CLD performance per device.

	True Floor	Test Number									
		1	2	3	4	5	6	7	8	9	10
D1	1	**1**,2	**1**,2	**1**,2	**1**,2	**1**,2	**1**,2	**1**,2	**1**,2	**1**,2	**1**,2
	2	1,**2**,3	1,**2**,3	1,**2**,3	1,**2**,3	1,**2**,3	1,**2**,3	1,**2**,3	1,**2**,3	1,**2**,3	1,**2**,3
	3	1,**2**,3	1,**2**,3	1,**2**,3	1,**2**,3	1,**2**,3	1,**2**,3	1,**2**,3	3,**4**,5	3,**4**,5	1,**2**,3
	4	3,**4**,5	3,**4**,5	3,**4**,5	3,**4**,5	3,**4**,5	3,**4**,5	3,**4**,5	4,**5**,6	3,**4**,5	4,**5**,6
	5	4,**5**,6	4,**5**,6	4,**5**,6	4,**5**,6	4,**5**,6	4,**5**,6	4,**5**,6	4,**5**,6	4,**5**,6	4,**5**,6
	6	5,**6**	5,**6**	5,**6**	5,**6**	5,**6**	5,**6**	5,**6**	5,**6**	5,**6**	5,**6**
D2	1	**1**,2	**1**,2	**1**,2	**1**,2	**1**,2	**1**,2	**1**,2	**1**,2	**1**,2	**1**,2
	2	1,**2**,3	1,**2**,3	1,**2**,3	1,**2**,3	1,**2**,3	1,**2**,3	1,**2**,3	1,**2**,3	1,**2**,3	1,**2**,3
	3	2,**3**,4	2,**3**,4	2,**3**,4	2,**3**,4	2,**3**,4	2,**3**,4	2,**3**,4	2,**3**,4	2,**3**,4	2,**3**,4
	4	3,**4**,5	3,**4**,5	3,**4**,5	3,**4**,5	3,**4**,5	3,**4**,5	3,**4**,5	3,**4**,5	3,**4**,5	3,**4**,5
	5	4,**5**,6	4,**5**,6	4,**5**,6	4,**5**,6	4,**5**,6	4,**5**,6	4,**5**,6	4,**5**,6	4,**5**,6	4,**5**,6
	6	5,**6**	5,**6**	5,**6**	5,**6**	5,**6**	5,**6**	5,**6**	5,**6**	5,**6**	5,**6**

**Table 4:** Total FLD performance per device.

	Floor	CLD Results	SCC Value	SCC Ratio
D1	1	1	0.85	1.6
		2	0.54	
	2	1	0.37	2.2
		2	0.81	
	**3**	**1**	**0.44**	1.6
		**2**	**0.48**	
		**3**	**0.76**	
	**4**	**4**	**0.65**	4.5
		**5**	**0.15**	
		**6**	**0.07**	
	5	4	0.38	1.4
		5	0.68	
		6	0.48	
	6	5	0.23	3.4
		6	0.79	
D2	1	1	0.75	4.9
		2	0.15	
	2	1	0.37	2.1
		2	0.80	
		3	0.31	
	3	2	0.54	1.4
		3	0.79	
		4	0.50	
	4	3	0.10	3.5
		4	0.73	
		5	0.21	
	5	4	0.32	1.6
		5	0.88	
		6	0.56	
	6	5	0.23	3.4
		6	0.79	

**Table 5:** Level detection change module delay.

	True Step Index	Estimated Step Index	Floor Change Completion Time (s)	Floor Fix Time from FLD (s)
2 ⟶ 3	177	178	164.22	164.82
3 ⟶ 4	363	357	212.17	208.02
4 ⟶ 5	539	541	312.15	313.77
5 ⟶ 2	697	698	486.72	487.27

**Table 6:** FLT performance in the multi-floor scenario.

	Mean (m)	RMSE (m)	Max (m)
2-1	1.82	4.13	4.26
3	1.14	1.80	3.66
4	1.58	3.12	3.97
5	0.96	1.20	3.00
2-2	1.17	1.63	2.10

## Data Availability

The data used to support the findings of this study will be available from the corresponding authors upon request.
